# Plasmonic Ag Nanoparticle-Loaded n-p Bi_2_O_2_CO_3_/α-Bi_2_O_3_ Heterojunction Microtubes with Enhanced Visible-Light-Driven Photocatalytic Activity

**DOI:** 10.3390/nano12091608

**Published:** 2022-05-09

**Authors:** Haibin Li, Xiang Luo, Ziwen Long, Guoyou Huang, Ligang Zhu

**Affiliations:** 1 College of Materials Science and Engineering, Changsha University of Science and Technology, Changsha 410114, China; coastllee@hotmail.com (H.L.); xiangl0926@163.com (X.L.); lzw19961020@163.com (Z.L.); hgyviny123viny@163.com (G.H.); 2 Guangxi Key Laboratory of Agricultural Resources Chemistry and Biotechnology, College of Chemistry and Food Science, Yulin Normal University, Yulin 537000, China

**Keywords:** α-Bi_2_O_3_, Bi_2_O_2_CO_3_, silver, heterojunction, microtube, photocatalysis

## Abstract

In this study, n-p Bi_2_O_2_CO_3_/α-Bi_2_O_3_ heterojunction microtubes were prepared via a one-step solvothermal route in an H_2_O-ethylenediamine mixed solvent for the first time. Then, Ag nanoparticles were loaded onto the microtubes using a photo-deposition process. It was found that a Bi_2_O_2_CO_3_/α-Bi_2_O_3_ heterostructure was formed as a result of the in situ carbonatization of α-Bi_2_O_3_microtubes on the surface. The photocatalytic activities of α-Bi_2_O_3_ microtubes, Bi_2_O_2_CO_3_/α-Bi_2_O_3_ microtubes, and Ag nanoparticle-loaded Bi_2_O_2_CO_3_/α-Bi_2_O_3_ microtubes were evaluated based on their degradation of methyl orange under visible-light irradiation (λ > 420 nm). The results indicated that Bi_2_O_2_CO_3_/α-Bi_2_O_3_ with a Bi_2_O_2_CO_3_ mass fraction of 6.1% exhibited higher photocatalytic activity than α-Bi_2_O_3_. Loading the microtubes with Ag nanoparticles significantly improved the photocatalytic activity of Bi_2_O_2_CO_3_/α-Bi_2_O_3_. This should be ascribed to the internal static electric field built at the heterojunction interface of Bi_2_O_2_CO_3_ and α-Bi_2_O_3_ resulting in superior electron conductivity due to the Ag nanoparticles; additionally, the heterojunction at the interfaces between two semiconductors and Ag nanoparticles and the local electromagnetic field induced by the surface plasmon resonance effect of Ag nanoparticles effectively facilitate the photoinduced charge carrier transfer and separation of α-Bi_2_O_3_. Furthermore, loading of Ag nanoparticles leads to the formation of new reactive sites, and a new reactive species ·O^2^^−^ for photocatalysis, compared with Bi_2_O_2_CO_3_/α-Bi_2_O_3_.

## 1. Introduction

In the past decades, photocatalytic technology through semiconductor oxides for the purification and treatment of polluted water and air has been extensively studied. Recent research activity in the field of heterogeneous photocatalysis is focused on exploiting novel and more efficient photocatalysts capable of using visible light for the degradation of organic contaminants. Many Bi-based semiconductors, such as BiVO_4_ [1], Bi_2_O_3_ [2], Bi_2_WO_6_ [3], Bi_2_O_2_CO_3_ [4], Bi_2_MoO_6_ [5], and BiPO_4_ [6] have been developed as visible-light-driven photocatalysts. Among them, Bi_2_O_3_ has received significant attention in recent years. It is well known that Bi_2_O_3_ is a p-type semiconductor with five crystallographic polymorphs denoted as monoclinic α-Bi_2_O_3_, tetragonal β-Bi_2_O_3_, cubic (BCC) γ-Bi_2_O_3_, cubic (FCC) δ-Bi_2_O_3_, and triclinic ω-Bi_2_O_3_ [2]. Monoclinic α-Bi_2_O_3_, which is nontoxic and chemically stable in aqueous solution under irradiation, has been proved to be a visible-light-driven photocatalyst, owing to its narrow band-gap energy (band gap around 2.6–2.8 eV). However, as a photocatalyst, α-Bi_2_O_3_ suffered severe problems in practical applications due to its low quantum yield, which is normally caused by the rapid recombination of its charge carriers [2]. Thus, novel photocatalysts based on α-Bi_2_O_3_ are required to be further explored in order to achieve increases in quantum efficiency and successes in practical applications.

Coupling a p-type α-Bi_2_O_3_ with another n-type semiconductor with matching band potentials to form a p-n heterojunction has been demonstrated to be an effective strategy to enhance the quantum yield. Driven by the internal static electric field built at the heterojunction interface, the photogenerated charges can transport from one semiconductor to another, thus improving the electron–hole pairs separation and interfacial charge transfer efficiency [7]. Bi_2_O_2_CO_3_ is an n-type semiconductor with a band gap of 3.55 eV. Growing attention has been paid to it, since Zhang et al. reported for the first time the application of Bi_2_O_2_CO_3_ as a photocatalyst in the degradation of methyl orange in aqueous solution under UV light irradiation [8]. Since α-Bi_2_O_3_ and Bi_2_O_2_CO_3_ are intrinsic p-type and n-type semiconductors, respectively; thus theoretically, an n-p Bi_2_O_2_CO_3_/α-Bi_2_O_3_ heterojunction is formed when the two dissimilar crystalline semiconductors combine. The reason for this is that the conduction band edge for α-Bi_2_O_3_ is much higher than that for Bi_2_O_2_CO_3_. As a well-defined interface is the key to improving the catalytic activities of heterojunction photocatalysts by facilitating charge transfer and separation, it is of great significance to develop a facile route to fabricate Bi_2_O_2_CO_3_/α-Bi_2_O_3_ heterostructures with effective contacts between Bi_2_O_2_CO_3_ and α-Bi_2_O_3_.

Noble metal nanoparticles (NPs), such as Au NPs [9,10], Pt NPs [11,12], Ru NPs [13,14], Ag NPs [15,16], and so on, have been used as co-catalysts to work with photocatalysts for enhanced photocatalytic performance, not only because they play the crucial roles of being photoinduced electron trappers due to their superior electron conductivities, but also because of the surface plasmon resonance (SPR) effect caused by the mutual oscillation between incident light and the electrons on the surface of noble metal NPs. Ag nanoparticles are a good choice for constructing noble metal NPs/semiconductor heterostructures, due to their facile preparation and relatively low cost. So far, several Ag NP-hybridized heterostructures have been reported, including Ag-Cu_2_O/PANI [17], Ag/ZnO@CF [18], Ag/AgCl/Ag_2_MoO_4_ [19], Ag/ZnO/3Dgraphene [20], Ag/GO/TiO_2_ [21], Bi_2_WO_6_/Ag_3_PO_4_-Ag [22], and g-C_3_N_4_/Ag/TiO_2_ [23], with enhanced photocatalytic activity. To the best of our knowledge, no study has been performed on synthesis and photocatalytic application of Ag NP-loaded Bi_2_O_2_CO_3_/α-Bi_2_O_3_ heterostructure composite systems.

In the present study, novel n-p Bi_2_O_2_CO_3_/α-Bi_2_O_3_ heterojunction microtubes with hexagonal cross sections were prepared via a facile one-step template- and surfactant-free solvothermal method for the first time. As Bi_2_O_2_CO_3_ was formed via in situ carbonatization of α-Bi_2_O_3_ microtubes on the surface, this method is more conducive to generate well-defined Bi_2_O_2_CO_3_/α-Bi_2_O_3_ heterojunction interfaces than two-step strategies. Co-catalyst Ag nanoparticles were evenly loaded on the surface of Bi_2_O_2_CO_3_/α-Bi_2_O_3_ heterojunction microtubes, using a photo-deposition process to construct a novel Ag/Bi_2_O_2_CO_3_/α-Bi_2_O_3_ microtube ternary system to further enhance the photocatalytic activity. The photocatalytic performances of the as-prepared samples were evaluated by examining the degradation of methyl orange (MO) under visible light (λ > 420 nm) irradiation.

## 2. Materials and Methods

### 2.1. Synthesis of Bi_2_O_2_CO_3_/α-Bi_2_O_3_ Heterostructure Microtubes

**Figure 1:**
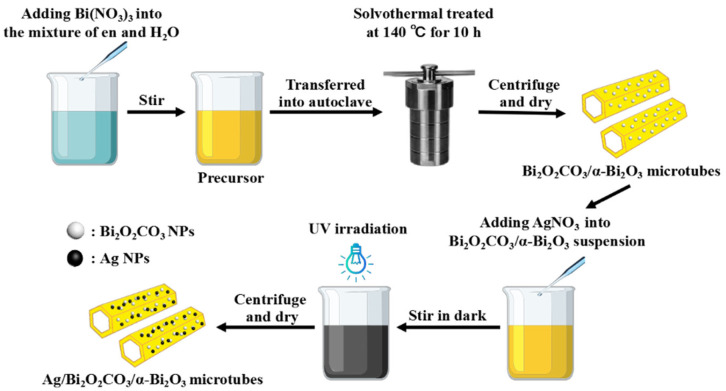
Schematic illustration for the synthesis of Ag NP-loaded Bi_2_O_2_CO_3_/α-Bi_2_O_3_ heterostructure microtubes.

Bismuth nitrate pentahydrate and ethylenediamine were purchased from Xilong Scientific Co., Ltd (Shantou, China) and Taicang Hushi Reagent Co., Ltd (Taicang, China), respectively. All reagents were of AR grade, and used without further purification. Distilled water was used in all experiments. As illustrated in Figure 1, in a typical synthesis, 0.00175 mol of Bi(NO_3_)_3_·5H_2_O was added into the ethylenediamine (en)–water mixture (80 mL), with a certain volume ratio of ethylenediamine and water (V_en_:V_water_). After being stirred for 30 min, the resulting faint yellow suspension (donated as precursor) was transferred into a 100-milliliter Teflon-lined stainless steel autoclave. The autoclave was sealed and maintained at 140 °C for 10 h and then cooled down to room temperature. The resulting precipitate was centrifuged, rinsed repeatedly with distilled water and ethanol, then dried at 80 °C in air to obtain the Bi_2_O_2_CO_3_/α-Bi_2_O_3_ heterostructure microtubes.

### 2.2. Synthesis of Ag NP-Loaded Bi_2_O_2_CO_3_/α-Bi_2_O_3_ Heterostructure Microtubes

The fabrication of Ag NP-loaded Bi_2_O_2_CO_3_/α-Bi_2_O_3_ heterostructure microtubes was conducted as follows. First, 0.5 g of Bi_2_O_2_CO_3_/α-Bi_2_O_3_ heterostructure microtubes was dispersed into the AgNO_3_ ((AR grade, Sinopharm Chemical Reagent Co., Ltd, Shanghai, China) aqueous solution under stirring. The theoretical loading amount of silver was set at 3 wt% in the Ag/Bi_2_O_2_CO_3_/α-Bi_2_O_3_ sample. After being ultrasonically treated for 10 min, the suspension was further magnetically stirred for 10 h in the dark, followed by UV illumination for 2 h under stirring. The black powder was centrifuged, rinsed with distilled water repeatedly to purify the product, and finally dried at 80 °C in air.

### 2.3. Characterization

The crystalline structure of the samples was analyzed by a Rigaku D/Max 2500 powder diffractometer (XRD) (Tokyo, Japan) with Cu Kα radiation (λ = 1.5406 Å). The morphology of the as-prepared samples was characterized by field-emission scanning electron microscopy (FESEM, FEI SIRION 200, Hillsboro, OR, USA), and transmission electron microscopy (TEM, Philips Tecnai 20 G2 S-TWIN, Hillsboro, OR, USA). X-ray photoelectron spectroscopy (XPS) data of the samples were determined with a K-Alpha 1063 electron spectrometer from Thermo Fisher Scientific (East Grinstead, West Sussex, UK) using 72W Al Kα radiation. Infrared spectroscopy analysis (IR) of the samples was performed on an AVATAR360 IR analyzer (Madison, WI, USA). UV-vis diffuse reflectance spectra (UV-vis) were measured with a Specord 200 UV spectrophotometer (Schönwalde-Glien, Germany).

### 2.4. Photocatalytic Experiments

The photocatalytic properties of the as-prepared samples were assessed by degradation of MO under the irradiation of visible light (λ > 420 nm). First, 0.5 g of photocatalyst was added to 100 mL of 10 mg/L MO aqueous solution. Then, the suspension was magnetically stirred in the dark for 1h before commencing the photocatalytic reactions, to allow the system to reach an adsorption/desorption equilibrium. All photocatalytic reactions were carried out in a laboratory constructed photo-reactor under visible light irradiation from a 500W Xe lamp equipped with a 420-nanometer cutoff filter. The photocatalytic system was magnetically stirred simultaneously during the course of illumination. At given time intervals, 3.5-milliliter aliquots of the aqueous solution were collected and centrifuged. The concentrations of MO solution were evaluated by measuring its absorption on a UNICO UV-2100 spectrophotometer (Palo Alto, CA, USA) at 463 nm, from which the photocatalytic activity was calculated.

## 3. Results and Discussion

**Figure 2:**
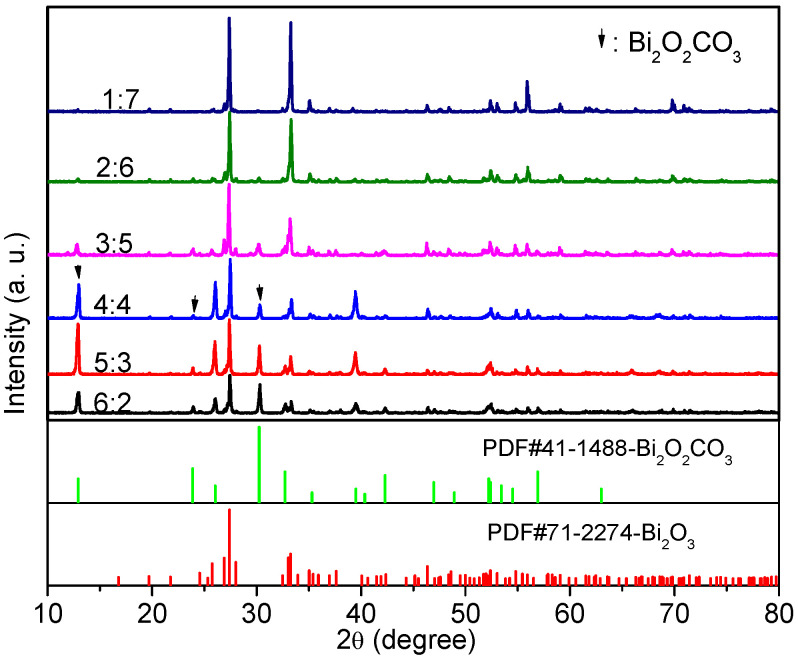
XRD patterns of the samples prepared at 140 °C for 10 h in the ethylenediamine–water mixture with various ratios of V_en_:V_water_.

XRD was used to analyze the phase composition and crystal structure of the samples. Figure 2 shows the XRD patterns of the samples produced at 140 °C for 10 h in the ethylenediamine–water mixture with various ratios of V_en_:V_water_. For all the samples, the diffraction peaks are sharp, and the intensity of the diffraction is high, indicating that the products are well-crystallized. In addition, the diffraction peaks assigned to α-Bi_2_O_3_ (JCPDS Card No. 71-2274) are accompanied by three characteristic peaks of Bi_2_O_2_CO_3_ (JCPDS Card No. 41-1488) at 12.9°, 23.8°, and 30.2°. No peaks of any additional phases were detected, indicating that the products exhibit a coexistence of both α-Bi_2_O_3_ and Bi_2_O_2_CO_3_ phases. Furthermore, when increasing the ratio of V_en_:V_water_, the intensity of the characteristic peaks attributed to Bi_2_O_2_CO_3_ gradually increases, whereas the intensity of the diffraction peaks assigned to α-Bi_2_O_3_ decreases. The mass fractions of the Bi_2_O_2_CO_3_ in the samples are 0%, 6.1%, 15.5%, 36.7%, 47.9%, and 51.3% for the samples prepared at V_en_:V_water_ ratios of 1:7, 2:6, 3:5, 4:4, 5:3, and 6:2, respectively, which were estimated from XRD intensity data by using the formula as expressed by Equation (1):

(1) $$R_{C}=\frac{I_{C}}{I_{C}+I_{O}}$$

where I_C_ and I_O_ are the integrated intensities of Bi_2_O_2_CO_3_ (013) and α-Bi_2_O_3_ (113) diffraction peaks, respectively. It can be inferred that the ratio of V_en_:V_water_ plays a key role in the phase composition of the products, and that a larger proportion of en favors the generation of Bi_2_O_2_CO_3_.

**Figure 3:**
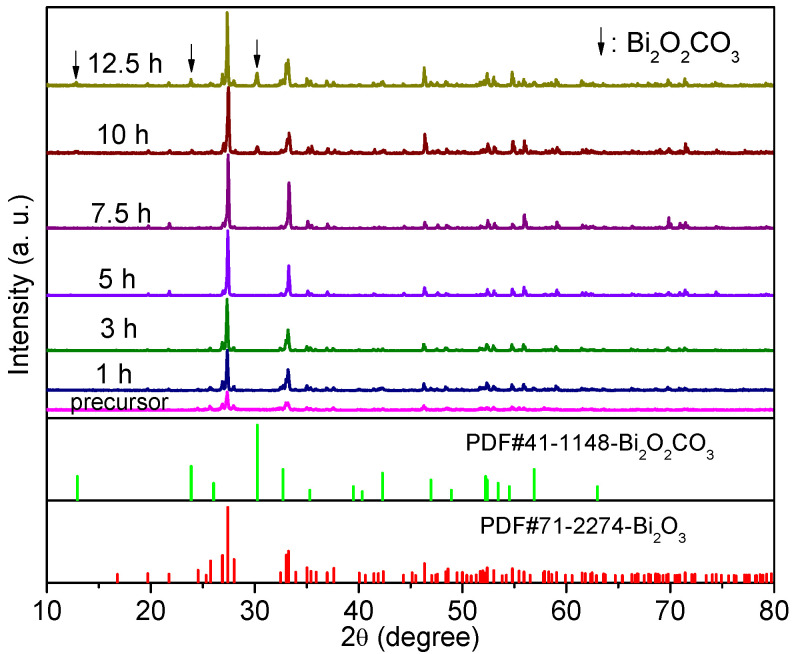
XRD patterns of the precursor and the samples obtained at 140 °C for 1, 3, 5, 7.5, 10 h, and 12.5 h in the ethylenediamine–water mixture with a V_en_:V_water_ ratio of 2:6.

How are the α-Bi_2_O_3_ and Bi_2_O_2_CO_3_ generated? Why does the proportion of en in the mixed solvent have such a significant effect on the generation of Bi_2_O_2_CO_3_? In order to answer these questions, XRD investigations on the precursor and the products obtained at 140 °C for 1, 3, 5, 7.5, 10, and 12.5 h in the en–water mixture with a V_en_:V_water_ ratio of 2:6 were carried out. The results are presented in Figure 3. For the precursor and the products obtained after solvothermal treatment for 1 h, 3 h, 5 h, and 7.5 h, all the diffraction peaks can be readily indexed to a pure α-Bi_2_O_3_ (JCPDS Card No. 71-2274) phase, revealing that α-Bi_2_O_3_ was formed before solvothermal treatment, and that a pure α-Bi_2_O_3_ phase could be maintained via controlling the reaction time using this technique. Moreover, the diffraction peaks of the solvothermal-treated products are much narrower than that of the precursor, and the peak intensities of the solvothermal-treated products are much higher, indicating that solvothermal treatment improved the crystallinity of the products. As the time increased to 10 h, the diffraction pattern of the sample indexed to the mixture of α-Bi_2_O_3_ and Bi_2_O_2_CO_3_ (JCPDS Card No. 41-1488). Three weak peaks at 12.9°, 23.8°, and 30.2° can be attributed to Bi_2_O_2_CO_3_. Further prolonging the time to 12.5 h, the intensity of the peaks indexed to Bi_2_O_2_CO_3_ increases, suggesting an increase in the amount of Bi_2_O_2_CO_3_. From the XRD results, it can be seen that the Bi_2_O_2_CO_3_/α-Bi_2_O_3_ composite is derived from α-Bi_2_O_3_, but not formed at the precursor stage.

**Figure 4:**
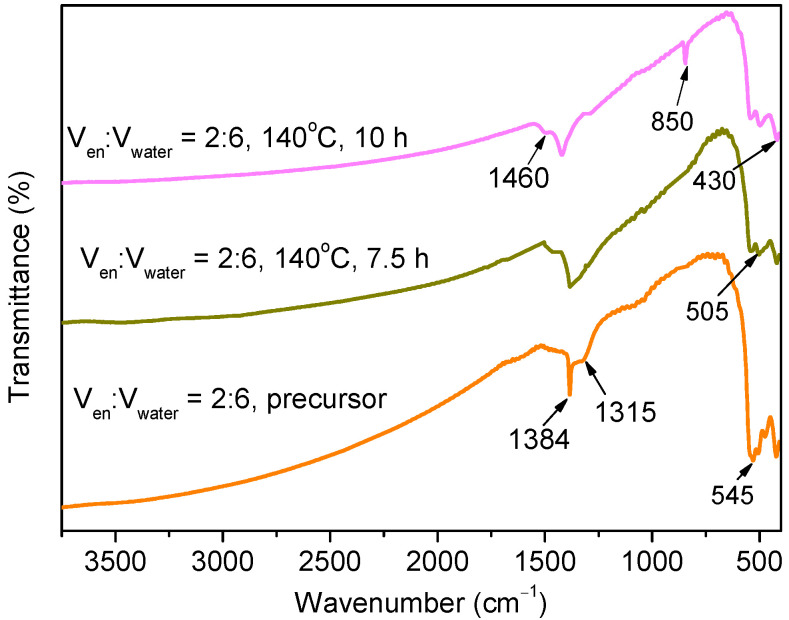
FT-IR spectra of the precursor and the samples obtained at 140 °C for 7.5 h and 10 h in the ethylenediamine–water mixture with a V_en_:V_water_ ratio of 2:6.

This is also supported by FT-IR spectra of the precursor and the products obtained after solvothermal treatment for 7.5 h and 10 h (Figure 4). For all the samples, the weak adsorptions at 1460, 1384, and 1315 cm^−1^ may be attributed to the carbonated species formed by the reactions between the surface hydroxyl groups and atmospheric CO_2_. The peaks at around 545, 505, and 430 cm^−1^ are due to the vibration of Bi-O bonds in BiO_6_ octahedral units [24,25]. It is necessary to mention that only the product obtained after solvothermal treatment for 10 h shows an extra band at 850 cm^−1^, which is ascribed to the CO_3_^2−^, indicating the formation of Bi_2_O_2_CO_3_ at this stage [24,25].

Based on the XRD and FT-IR analyses, formation of the Bi_2_O_2_CO_3_/α-Bi_2_O_3_ composite in the present solvothermal process could be described by following reactions:

(2) $$H_{2}NCH_{2}CH_{2}NH_{2}+2H_{2}O\to H_{3}\overset{+}{N}CH_{2}CH_{2}\overset{+}{N}H_{3}+2OH^{-}$$

(3) $$Bi^{3+}+3OH^{-}\to Bi(OH{)}_{3}↓$$

(4) $$2Bi{\left(OH\right)}_{3}\to Bi_{2}O_{3}+3H_{2}O$$

(5) $$CO_{2}+2OH^{-}\to CO_{3}{}^{2-}+H_{2}O$$

(6) $$Bi_{2}O_{3}+CO_{3}{}^{2-}+H_{2}O\to Bi_{2}O_{2}CO_{3}+2OH^{-}$$

When Bi(NO_3_)_3_·5H_2_O was added to the en–water mixture with a V_en_:V_water_ ratio of 2:6, the reaction was performed in a strong alkali condition, as indicated in Equation (2). Abundant hydroxide ions firstly reacted with Bi^3+^ to produce Bi(OH)_3_, which then dehydrated to form α-Bi_2_O_3_ under vigorous stirring, as illustrated in Equations (3) and (4). Due to the presence of en, the mixed solvent easily captured CO_2_ from the air to generate CO_3_^2−^ before being transferred into the autoclave. In prolonging the solvothermal treatment time to 10 h, a small amount of obtained α-Bi_2_O_3_ reacted with CO_3_^2−^ in the solvent to give rise to Bi_2_O_2_CO_3_, as summarized in Equations (5) and (6) [26]. It can be concluded that Bi_2_O_2_CO_3_ was formed by in situ carbonatization of α-Bi_2_O_3_. A larger proportion of en in the solvent captures more CO_2_ to generate more CO_3_^2−^, resulting in a higher ratio of Bi_2_O_2_CO_3_ in the product.

**Figure 5:**
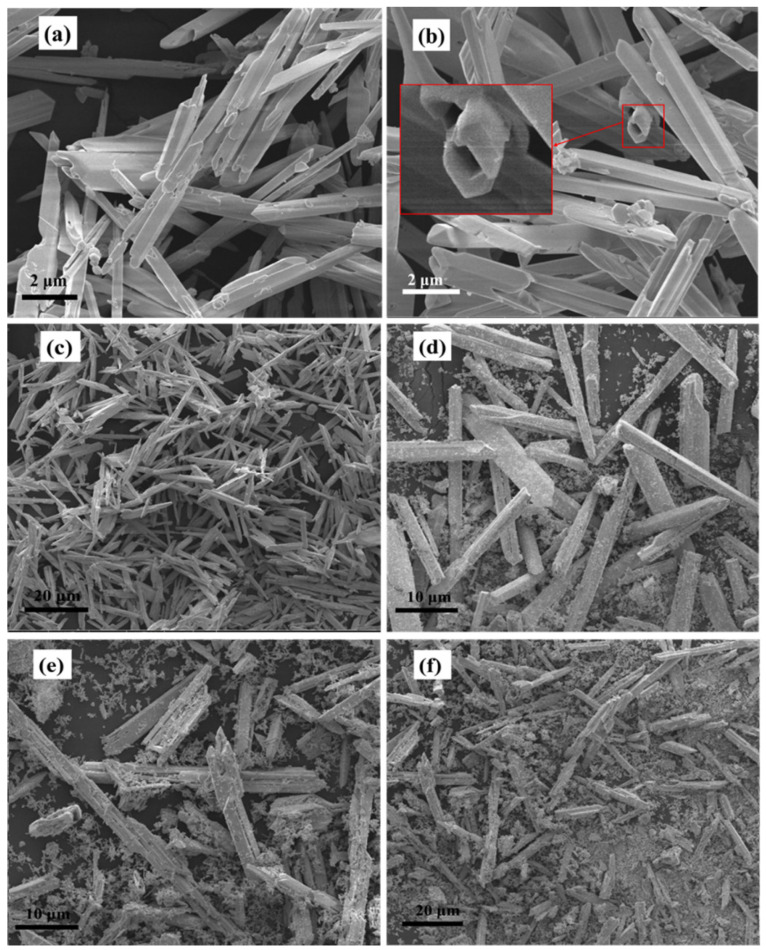
SEM images of the samples prepared at 140 °C for 10 h in the en–water mixture with various ratios of V_en_:V_water_: (**a**) 1:7, (**b**,**c**) 2:6, (**d**) 4:4, (**e**) 5:3, and (**f**) 6:2.

Figure 5a,b show the SEM images of the products obtained by solvothermal treatment at 140 °C for 10 h in the ethylenediamine–water mixture with V_en_:V_water_ ratios of 1:7 and 2:6, respectively. It can be seen that both samples consist of microtubes. The magnified image of the microtubes presented in the left insert of Figure 5b clearly demonstrates that the microtubes have well-defined hexagonal cross sections. The SEM image with low magnification (Figure 5c) reveals that the products obtained in the ethylenediamine–water mixture with a V_en_:V_water_ ratio of 2:6 are almost entirely microtubes with lengths of 5–30 μm, and side lengths of 0.2–1 μm, indicating the high yield of microtubes in this condition. However, when the V_en_:V_water_ ratio was controlled at 4:4, 5:3, and 6:2, the as-prepared products contain microtubes and a lot of irregular particles, as presented in Figure 5d–f, respectively. This indicates that the proportion of en in the mixed solvent also has a significant effect on the morphology of the products. More en in the solvent captures more CO_2_ to generate more CO_3_^2−^, which makes more α-Bi_2_O_3_ carbonatized, resulting in the destruction of microtubes.

**Figure 6:**
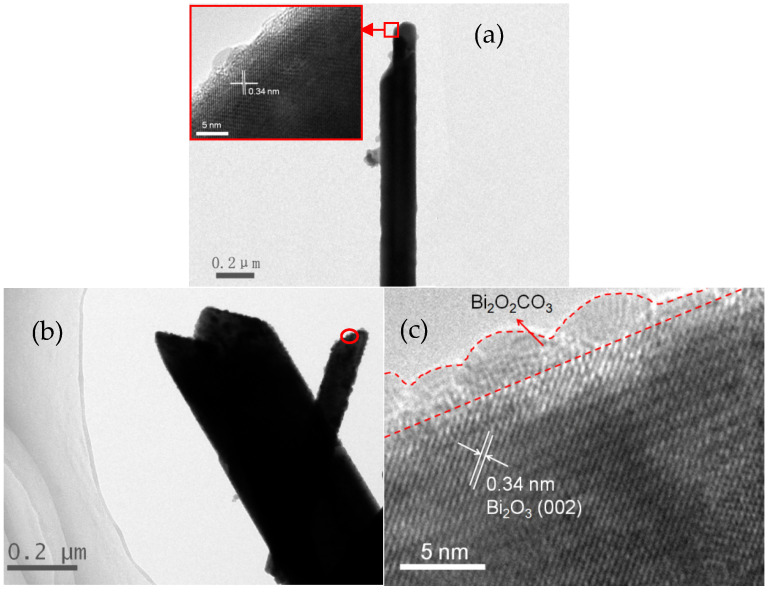
TEM images of (**a**) α-Bi_2_O_3_ microtubes (insert: HRTEM) and (**b**) Bi_2_O_2_CO_3_/α-Bi_2_O_3_ microtubes; an HRTEM image of (**c**) Bi_2_O_2_CO_3_/α-Bi_2_O_3_ microtubes.

Figure 6a presents the TEM image of the obtained α-Bi_2_O_3_ microtube prepared at a V_en_:V_water_ ratio of 2:6 for 7.5 h. There is a contrast between the inner and outside parts of the sample, confirming its tubular structure. The lattice spacing of about 0.34 nm between adjacent lattice planes in the insert corresponds to the interplanar spacing of the (002) plane of α-Bi_2_O_3_. Figure 6b shows the TEM image of Bi_2_O_2_CO_3_/α-Bi_2_O_3_ heterojunction microtubes prepared at a V_en_:V_water_ ratio of 2:6 for 10 h. It can be clearly seen that a lot of nanoparticles highly disperse on the surface of α-Bi_2_O_3_ microtubes, which are considered to be Bi_2_O_2_CO_3_ particles. No “support-free” Bi_2_O_2_CO_3_ nanoparticles are found, indicating that those nanoparticles are strongly anchored to the α-Bi_2_O_3_ microtubes. From the HRTEM image of the sample shown in Figure 6c, it can be seen that the lattice structure of α-Bi_2_O_3_ is very orderly and different from that of Bi_2_O_2_CO_3_ nanoparticles. The measured lattice fringes of 0.34 nm well match the (002) crystallographic planes of α-Bi_2_O_3_. In particular, it can be well confirmed that the Bi_2_O_2_CO_3_ nanoparticles are anchored on the surface of the α-Bi_2_O_3_ substrate, forming a good attachment. The obvious interface between the Bi_2_O_2_CO_3_ nanoparticles and the α-Bi_2_O_3_ microtubes shown in HRTEM images implies the formation of a well-defined heterojunction structure. Because α-Bi_2_O_3_ and Bi_2_O_2_CO_3_ are p-type and n-type semiconductors, respectively, the heterojunction can be considered to be a well-defined and well-formed p–n junction.

**Figure 7:**
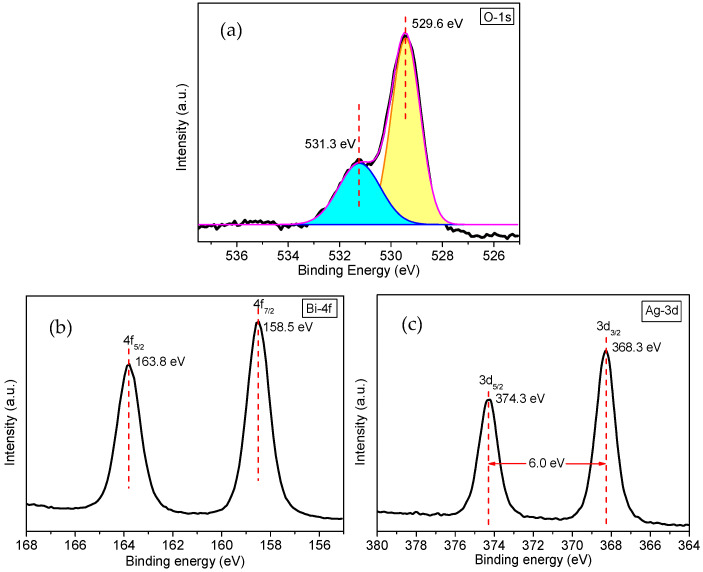
High-resolution XPS spectra of (**a**) O 1s, (**b**) Bi 4f, and (**c**) Ag 3d.

Figure 7 shows the high-resolution XPS spectra of Bi, O, and Ag in Ag NP-loaded Bi_2_O_2_CO_3_/α-Bi_2_O_3_ heterojunction microtubes with R_c_ of 6.1%. As observed in the XPS spectrum of Bi 4f (Figure 7a), two strong peaks at 163.8 and 158.5 eV are assigned to Bi 4f_5/2_ and Bi 4f_7/2_, respectively, confirming that the bismuth species in the sample are Bi^3+^ cations [27]. In the O 1s XPS spectrum (Figure 7b), the O 1s region is fitted by two peaks at 529.6 and 531.3 eV, which are attributed to the oxygen in the Bi–O bond and carbonate species, respectively [27]. Figure 7c presents the Ag 3d XPS spectrum, with two peaks at 368.3 and 374.3 eV, which correspond to Ag 3d_5/2_ and Ag 3d_3/2_, respectively, suggesting that the silver species in the sample is metallic silver, as the bonding energy corresponding to Ag 3d_5/2_ of metallic Ag and Ag_2_O are 368.25 eV and 367.70 eV, respectively, according to the previous report [28].

**Figure 8:**
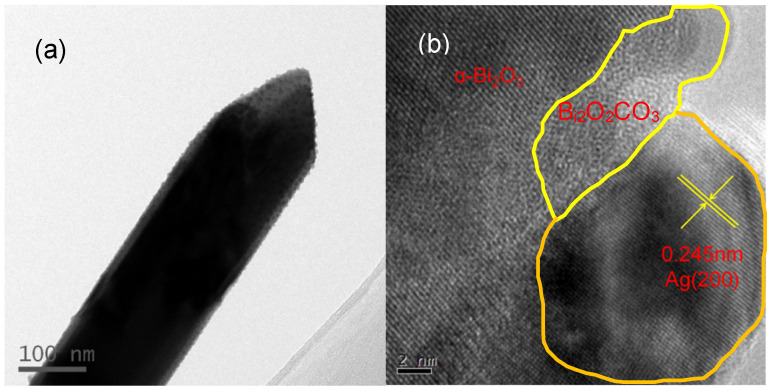
TEM (**a**) and HRTEM (**b**) images of Ag-loaded Bi_2_O_2_CO_3_/α-Bi_2_O_3_ heterojunction microtube.

The TEM image of Ag NP-loaded Bi_2_O_2_CO_3_/α-Bi_2_O_3_ heterojunction microtubes with R_c_ of 6.1% is shown in Figure 8a. As seen from the image, many nanoparticles are evenly dispersed on the surface of microtubes, and strongly anchored. HRTEM was carried out to verify the nanoparticles, as shown in Figure 8b. The lattice structure of nanoparticles anchored on the surface of microtubes is very orderly, and obviously different from that of the microtubes. The measured lattice fringes of 0.245 nm well match the (200) crystallographic planes of metallic Ag, suggesting that Ag NP-loaded Bi_2_O_2_CO_3_/α-Bi_2_O_3_ heterojunction microtubes are achieved by this strategy.

**Figure 9:**
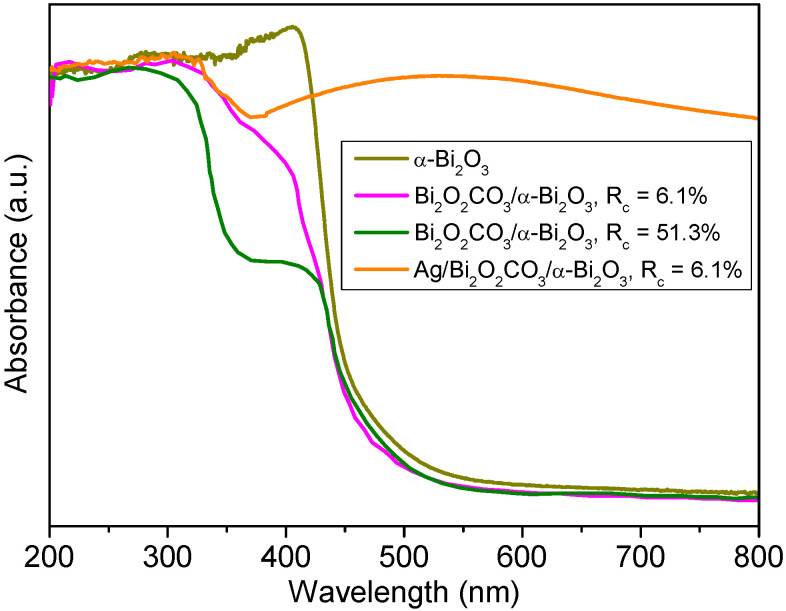
UV−vis diffuse reflectance spectra of α-Bi_2_O_3_, Bi_2_O_2_CO_3_/α-Bi_2_O_3_, and Ag/Bi_2_O_2_CO_3_/α-Bi_2_O_3_.

Figure 9 shows the UV−vis diffuse reflectance spectra of α-Bi_2_O_3_ microtubes, Bi_2_O_2_CO_3_/α-Bi_2_O_3_ heterojunction microtubes, and Ag NP-loaded Bi_2_O_2_CO_3_/α-Bi_2_O_3_ heterojunction microtubes. The α-Bi_2_O_3_ microtubes prepared at V_en_:V_water_ = 1:7 exhibit strong absorption in the visible range in addition to the UV range. The absorption edge occurs at about 450 nm. The spectrum is steep, indicating that the absorption of visible light is not due to the transition from impurity levels, but to the band-gap transition. The Bi_2_O_2_CO_3_/α-Bi_2_O_3_ heterojunction microtubes with R_c_ of 6.1% and 51.3% show dual absorption edges at 365 and 450 nm, which are related to their mixed-phase structure. Moreover, the absorbance in the 360–450 nm range of Bi_2_O_2_CO_3_/α-Bi_2_O_3_ is much weaker compared with that of α-Bi_2_O_3_ due to the its substantial Bi_2_O_2_CO_3_ phase content. The band-gap energies were estimated to be 2.75 and 3.4 eV for α-Bi_2_O_3_ and Bi_2_O_2_CO_3_, respectively, and were calculated from the formula λ_g_ = 1239.8/E_g_, where λ_g_ is the band-gap wavelength, and E_g_ is the bandgap energy [29]. Ag NP-loaded Bi_2_O_2_CO_3_/α-Bi_2_O_3_ heterojunction microtubes with R_c_ of 6.1% show an extended absorption in the visible region, which is due to the typical surface plasmon band exhibited by the Ag nanoparticles [30].

**Figure 10:**
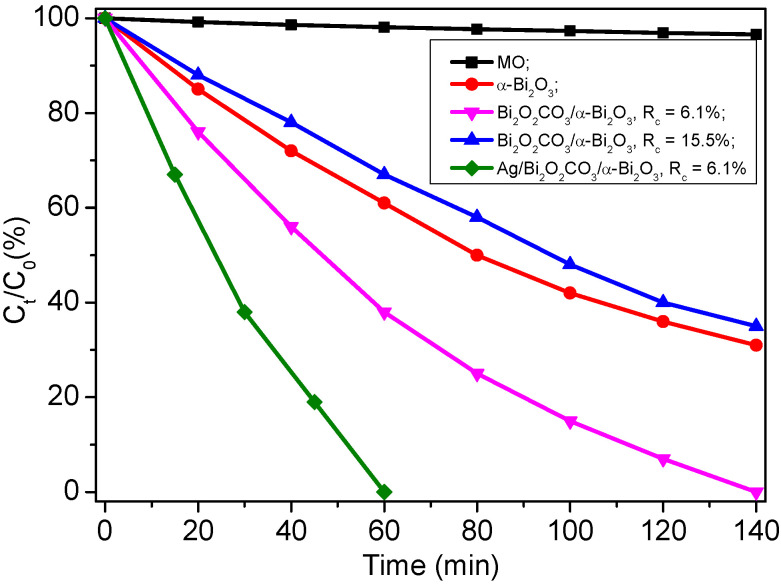
The residual MO at different irradiation time for the as-prepared samples.

Photodegradation of MO under visible light irradiation was carried out to estimate the photocatalytic performance of the as-prepared samples. The photodegradation efficiencies of MO as a function of irradiation time by α-Bi_2_O_3_, Bi_2_O_2_CO_3_/α-Bi_2_O_3_ with R_c_ of 6.7%, Bi_2_O_2_CO_3_/α-Bi_2_O_3_ with R_c_ of 15.5%, Ag/Bi_2_O_2_CO_3_/α-Bi_2_O_3_ with R_c_ of 6.7%, as well as in the absence of photocatalysts, are presented in Figure 10. It can be seen that all the samples show visible light photocatalytic activities. After 140 min of irradiation, the photodegradation efficiencies of MO by α-Bi_2_O_3_, Bi_2_O_2_CO_3_/α-Bi_2_O_3_ with R_c_ of 6.7%, and Bi_2_O_2_CO_3_/α-Bi_2_O_3_ with R_c_ of 15.5%, reach 69%, 100%, and 65%, respectively. For Ag/Bi_2_O_2_CO_3_/α-Bi_2_O_3_ with R_c_ of 6.7%, it reaches 100% after 60 min. Generally, the overall photocatalytic activity of a semiconductor is primarily dictated by surface area, photoabsorption ability, and the separation and transporting rates of photoinduced electron/hole pairs in the catalysts [31]. Since α-Bi_2_O_3_, Bi_2_O_2_CO_3_/α-Bi_2_O_3_ with R_c_ of 6.7%, and Ag/Bi_2_O_2_CO_3_/α-Bi_2_O_3_ possess similar size and morphology, the enhanced photocatalytic activities of Ag/Bi_2_O_2_CO_3_/α-Bi_2_O_3_ and Bi_2_O_2_CO_3_/α-Bi_2_O_3_ with R_c_ of 6.7% should be ascribed to the improved separation and transporting rates of photoinduced electron/hole pairs.

**Figure 11:**
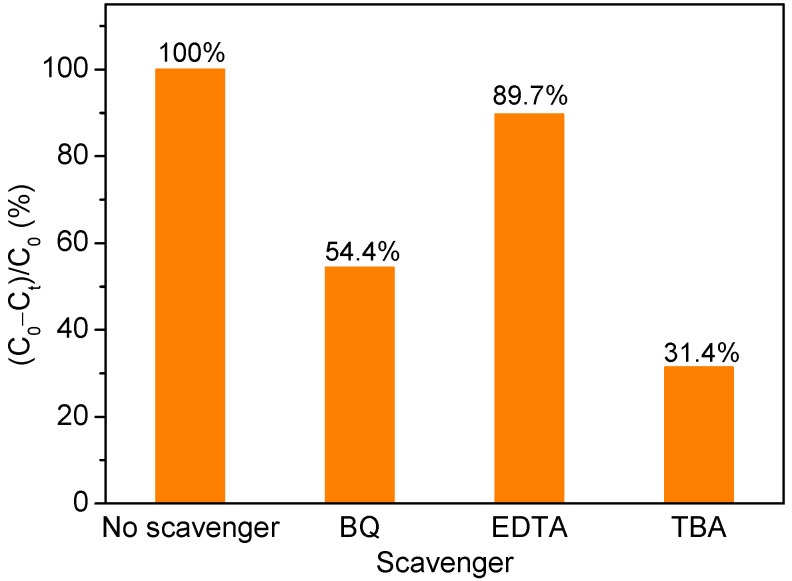
The photodegradation rates of MO by Ag/Bi_2_O_2_CO_3_/α-Bi_2_O_3_ after 60 min in the presence of various scavengers.

Photogenerated electrons, holes, ·O_2_^−^, and ·OH are considered to be major reactive species in organics photodegradation [32]. MO can be degraded into CO_2_, H_2_O, and other products by those reactive species [33]. In order to clarify the reaction mechanism further, 1 mmol of various scavengers was introduced to explore the specific reactive species that might play important roles in MO degradation by Ag/Bi_2_O_2_CO_3_/α-Bi_2_O_3_. Benzoquinone (BQ), ethylene diaminetetraacetic acid (EDTA), and tertiary butanol (TBA) were used as the scavengers for ·O_2_^−^, holes, and ·OH, respectively [34]. Figure 11 shows the photodegradation efficiencies of MO by Ag/Bi_2_O_2_CO_3_/α-Bi_2_O_3_ in the presence of these scavengers under visible light irradiation for 60 min. Both BQ and TBA show suppression of the degradation rate of MO, with TBA exhibiting a stronger suppressing effect. Meanwhile, EDTA shows a much weaker suppressing effect than BQ and TBA, suggesting that ·OH and ·O_2_^−^ are the major reactive species responsible for the photodegradation of MO by Ag/Bi_2_O_2_CO_3_/α-Bi_2_O_3_.

**Figure 12:**
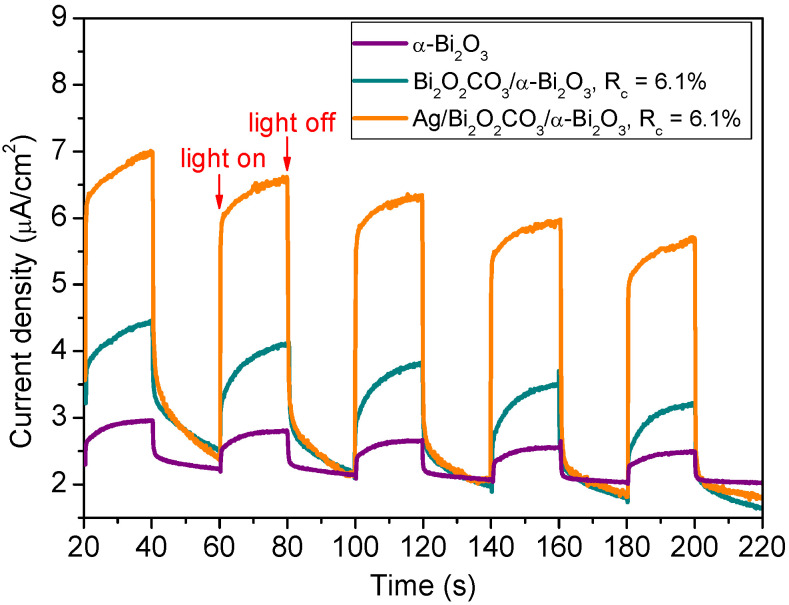
Photocurrent responses of different samples under visible light.

The effects of Bi_2_O_2_CO_3_/α-Bi_2_O_3_ and Ag NPs on the efficiency of photoinduced electrons and holes separation were investigated by the photocurrent tests, as shown in Figure 12. The photocurrent intensities of the samples follow the order of Ag/Bi_2_O_2_CO_3_/α-Bi_2_O_3_ > Bi_2_O_2_CO_3_/α-Bi_2_O_3_ > α-Bi_2_O_3_. As demonstrated in the previous research, higher photocurrent intensity means higher separation efficiency of the photoinduced electron/hole pairs. The photocurrent measurement results suggest that the formation of Bi_2_O_2_CO_3_/α-Bi_2_O_3_ heterostructures improves charge carrier transfer and separation of α-Bi_2_O_3_, while loading of Ag NPs on the heterostructures further enhances this effect. It is consistent with the photocatalytic performance.

**Figure 13:**
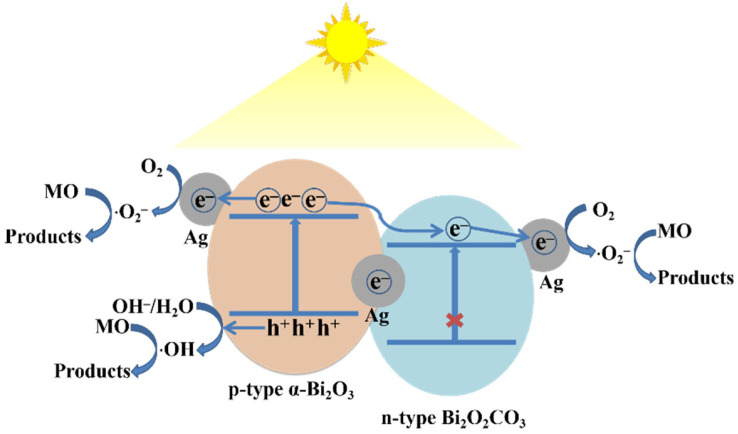
Schematic illustration of the proposed possible mechanism for photodegradation of MO by Ag/Bi_2_O_2_CO_3_/α-Bi_2_O_3_ under visible light irradiation.

According to the experimental results, we believe that there are four major reasons responsible for the enhanced photodegradation of MO by Ag NP-loaded Bi_2_O_2_CO_3_/α-Bi_2_O_3_ heterojunction microtubes, as illustrated in Figure 13. Firstly, Bi_2_O_2_CO_3_/α-Bi_2_O_3_ heterojunction facilitates the charge separation. As reported in the previous work, α-Bi_2_O_3_ is a p-type semiconductor, while Bi_2_O_2_CO_3_ is determined as an n-type material. The conduction band edge of α-Bi_2_O_3_ and Bi_2_O_2_CO_3_ at the point of zero charge (pH_zpc_) can be theoretically predicted from the formula E_CB_^0^ = X − E_c_ − 0.5E_g_, where X is the absolute electronegativity of the semiconductor, and E_c_ is the energy of free electrons on the hydrogen scale (4.5 eV) [35]. The values of X are 5.95 eV for α-Bi_2_O_3_ and 6.35 eV for Bi_2_O_2_CO_3_, while the estimated E_g_ is 2.75 eV for α-Bi_2_O_3_ and 3.4 eV for Bi_2_O_2_CO_3_. Given the formula above, the calculated E_CB_ and E_VB_ values are 0.075 eV and 2.825 eV for α-Bi_2_O_3_, respectively, and 0.15 eV and 3.55 eV for Bi_2_O_2_CO_3_, respectively. Therefore, both the conduction band (CB) and valence band (VB) of Bi_2_O_2_CO_3_ are considered to be at lower levels than those of α-Bi_2_O_3_. Thus, a Type II p-n heterojunction is formed at the interfaces as Bi_2_O_3_ and Bi_2_O_2_CO_3_ are closely joined together. When Bi_2_O_2_CO_3_/α-Bi_2_O_3_ heterojunction microtubes are exposed to visible light irradiation, the electrons in the VB of α-Bi_2_O_3_ are excited to its CB, leaving holes in the VB. However, for Bi_2_O_2_CO_3_, the electrons in the VB cannot be excited because of the wide bandgap of 3.4 eV. Due to the internal field resulting from the potential of band energy difference between α-Bi_2_O_3_ and Bi_2_O_2_CO_3_, there is a great tendency for α-Bi_2_O_3_ to transfer its photoexcited electrons into the CB of Bi_2_O_2_CO_3_, facilitating electron-hole separation in α-Bi_2_O_3_, and providing more holes for photocatalytic reactions. Secondly, as the Ag NPs loaded on the surface of Bi_2_O_2_CO_3_/α-Bi_2_O_3_ heterojunction microtubes are in close contact with α-Bi_2_O_3_ or Bi_2_O_2_CO_3_, the electrons in the CB of α-Bi_2_O_3_ and Bi_2_O_2_CO_3_ will transfer to the Ag NPs because of the superior electron conductivity of Ag NPs, along with the formation of heterojunctions at the interface between two semiconductors and the Ag NPs as a result of their work function differences, further suppressing charge carrier recombination [30]. Thirdly, as mentioned above, the valence bands of α-Bi_2_O_3_ are located at a deep position of about 2.825 eV versus NHE, which is more positive than that of ·OH/OH^−^ (1.9 eV vs. NHE), indicating that the photogenerated holes in the VB of α-Bi_2_O_3_ can react with OH^−^ to produce ·OH for oxidation of MO [35,36]. Meanwhile, the conduction band potentials of α-Bi_2_O_3_ and Bi_2_O_2_CO_3_ are close to +0.075 eV and +0.15 eV versus NHE, respectively, which are more positive than that of O_2_/·O_2_^−^ (−0.33 eV vs. NHE). Thus, it is impossible for the adsorption oxygen to capture an electron from the conduction bands of α-Bi_2_O_3_ and Bi_2_O_2_CO_3_ to form active oxygen species (·O_2_^−^) [35,36]. However, the electrons transferred to Ag NPs from the CBs of α-Bi_2_O_3_ and Bi_2_O_2_CO_3_ in Ag/Bi_2_O_2_CO_3_/α-Bi_2_O_3_ might be trapped by oxygen molecules in the solutions to form ·O_2_^−^ for reaction [30,35,36]. This means that loading Ag NPs onto the surface of Bi_2_O_2_CO_3_/α-Bi_2_O_3_ can bring another benefit that leads to the formation of new reaction active sites, and a new reactive species ·O_2_^−^, enhancing the photocatalytic activity of Bi_2_O_2_CO_3_/α-Bi_2_O_3_. The possible reactions in the Ag/Bi_2_O_2_CO_3_/α-Bi_2_O_3_ ternary photocatalytic system are illustrated by the following equations:

(7) $$Bi_{2}O_{3}+hv\to Bi_{2}O_{3}(h^{+}+e^{-})$$

(8) $$Bi_{2}O_{3}(e^{-})+Bi_{2}O_{2}CO_{3}\to Bi_{2}O_{3}+Bi_{2}O_{2}CO_{3}(e^{-})$$

(9) $$Bi_{2}O_{3}(e^{-})+Ag\to Bi_{2}O_{3}+Ag(e^{-})$$

(10) $$Bi_{2}O_{2}CO_{3}(e^{-})+Ag\to Bi_{2}O_{2}CO_{3}+Ag(e^{-})$$

(11) $$Bi_{2}O_{3}(h^{+})+OH^{-}\to Bi_{2}O_{3}+\cdot OH$$

(12) $$Ag(e^{-})+O_{2}\to Ag+\cdot O_{2}{}^{-}$$

(13) $$\cdot OH/\cdot O_{2}{}^{{}_{-}}+MO\to Product$$

Lastly, the surface plasmon resonance effect caused by the mutual oscillation between incident light and the electrons on the surface of metallic Ag NPs causes the rise of a local electromagnetic field [35]. Under the influence of this local electromagnetic field, the photogenerated electron/hole pairs on the α-Bi_2_O_3_ surface are effectively separated, which also enhances photocatalytic activity.

**Figure 14:**
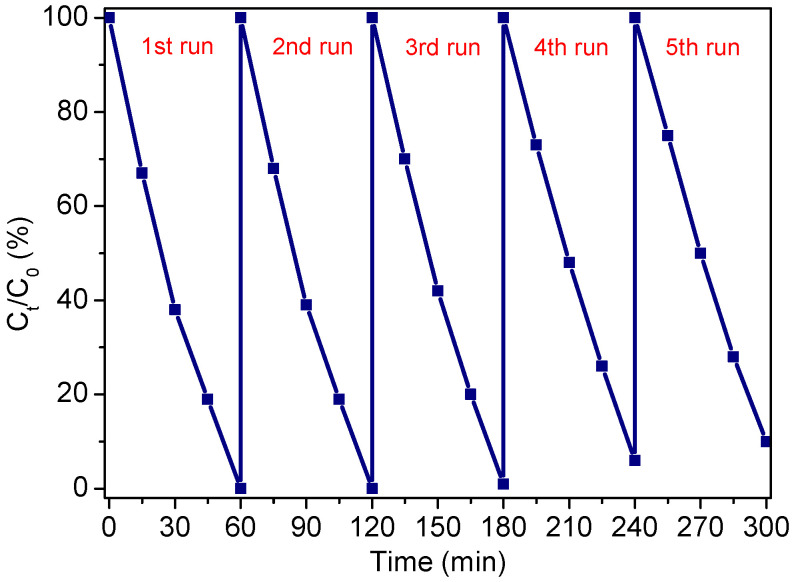
Cyclic photodegradation curve for Ag/Bi_2_O_2_CO_3_/α-Bi_2_O_3_.

Figure 14 presents the results of repeated experiments on photodegradation of MO by Ag/Bi_2_O_2_CO_3_/α-Bi_2_O_3_ under visible light irradiation. After each run, the photocatalysts were collected by centrifugation, followed by ultrasonic cleaning with distilled water. As shown in the image, no significant loss is found after four successive cycles; 89.8% of MO was degraded in the fifth run after 60 min of visible light irradiation, suggesting that the sample is stale and not photo-corroded in the photocatalytic reactions.

## 4. Conclusions

In summary, we have developed a facile solvothermal approach to prepare n-p Bi_2_O_2_CO_3_/α-Bi_2_O_3_ heterojunction microtubes. Plasmonic Ag NPs were loaded onto the Bi_2_O_2_CO_3_/α-Bi_2_O_3_ microtubes using a simple photo-deposition process, to construct an Ag/Bi_2_O_2_CO_3_/α-Bi_2_O_3_ ternary photocatalytic system. This Ag/Bi_2_O_2_CO_3_/α-Bi_2_O_3_ ternary system showed much higher photocatalytic activity than α-Bi_2_O_3_ and Bi_2_O_2_CO_3_/α-Bi_2_O_3_. Under visible light irradiation, the well-defined interfaces between Bi_2_O_2_CO_3_ and α-Bi_2_O_3_ in the heterojunctions due to the in situ carbonation of α-Bi_2_O_3_ on the surface into Bi_2_O_2_CO_3_, facilitate the transfer of photoinduced electrons from the CB of α-Bi_2_O_3_ to that of Bi_2_O_2_CO_3_. Meanwhile, the superior electron conductivity of Ag NPs, the heterojunction at the interface between two semiconductors and Ag NPs, and the local electromagnetic field induced by the surface plasmon resonance effect of Ag NPs, further promote the transfer of photoinduced electrons and suppress the recombination of hole/electron pairs, leaving more holes in the VB of α-Bi_2_O_3_ to produce more ·OH for photodegradation of MO. After the photoinduced electrons in the CB of α-Bi_2_O_3_ and Bi_2_O_2_CO_3_ that cannot form ·O_2_^−^ are transferred to Ag NPs, they combine with O_2_ to form ·O_2_^−^, which means that loading of Ag NPs onto Bi_2_O_2_CO_3_/α-Bi_2_O_3_ creates new reaction active sites and a new reactive species ·O_2_^−^ for photocatalysis, compared with Bi_2_O_2_CO_3_/α-Bi_2_O_3_.

## Data Availability

Data are contained within the article.
